# Comparing outcomes of out-of-hospital cardiac arrest patients with initial shockable rhythm in Singapore and Osaka using population-based databases

**DOI:** 10.1186/s13054-023-04771-5

**Published:** 2023-12-06

**Authors:** Yohei Okada, Nur Shahidah, Yih Yng Ng, Michael Y. C. Chia, Han Nee Gan, Benjamin S. H. Leong, Desmond R. Mao, Wei Ming Ng, Nausheen Edwin, Takeyuki Kiguchi, Norihiro Nishioka, Tetsuhisa Kitamura, Taku Iwami, Marcus Eng Hock Ong

**Affiliations:** 1 Duke-NUS Medical School, Health Services and Systems Research, Singapore, Singapore; 2 Department of Preventive Services, Graduate School of Medicine, Kyoto University, Kyoto, Japan; 3 Department of Emergency Medicine, Singapore General Hospital, Singapore, Singapore; 4 Lee Kong Chian School of Medicine, Nanyang Technological University, Singapore, Singapore; 5 Department of Preventive and Population Medicine, Tan Tock Seng Hospital, Singapore, Singapore; 6 Emergency Department, Tan Tock Seng Hospital, Singapore, Singapore; 7 Accident and Emergency, Changi General Hospital, Singapore, Singapore; 8 Emergency Medicine Department, National University Hospital, Singapore, Singapore; 9 Department of Acute and Emergency Care, Khoo Teck Puat Hospital, Singapore, Singapore; 10 Emergency Medicine Department, Ng Teng Fong General Hospital, Singapore, Singapore; 11 Department of Emergency Medicine, Sengkang General Hospital, Singapore, Singapore; 12 Division of Environmental Medicine and Population Sciences, Department of Social and Environmental Medicine, Graduate School of Medicine, Osaka University, Osaka, Japan; 13 Department of Emergency and Critical Care, Osaka General Medical Center, Osaka, Japan

## Abstract

**Background:**

Previous research indicated outcomes among refractory out-of-hospital cardiac arrest (OHCA) patients with initial shockable rhythm were different in Singapore and Osaka, Japan, possibly due to the differences in access to extracorporeal cardiopulmonary resuscitation. However, this previous study had a risk of selection bias. To address this concern, this study aimed to evaluate the outcomes between Singapore and Osaka for OHCA patients with initial shockable rhythm using only population-based databases.

**Methods:**

This was a secondary analysis of two OHCA population-based databases in Osaka and Singapore, including adult OHCA patients with initial shockable rhythm. A machine-learning-based prediction model was derived from the Osaka data (*n* = 3088) and applied to the PAROS-SG data (*n* = 2905). We calculated the observed-expected ratio (OE ratio) for good neurological outcomes observed in Singapore and the expected derived from the data in Osaka by dividing subgroups with or without prehospital ROSC.

**Results:**

The one-month good neurological outcomes in Osaka and Singapore among patients with prehospital ROSC were 70% (791/1,125) and 57% (440/773), and among patients without prehospital ROSC were 10% (196/1963) and 2.8% (60/2,132). After adjusting patient characteristics, the outcome in Singapore was slightly better than expected from Osaka in patients with ROSC (OE ratio, 1.067 [95%CI 1.012 to 1.125]), conversely, it was worse than expected in patients without prehospital ROSC (OE ratio, 0.238 [95%CI 0.173 to 0.294]).

**Conclusion:**

This study showed the outcomes of OHCA patients without prehospital ROSC in Singapore were worse than expected derived from Osaka data even using population-based databases.

(249/250 words).

**Supplementary Information:**

The online version contains supplementary material available at 10.1186/s13054-023-04771-5.

## Introduction

Extracorporeal cardiopulmonary resuscitation (ECPR), which utilizes extracorporeal membrane oxygenation (ECMO) during cardiopulmonary resuscitation, is an advanced procedure designed for OHCA patients who are unresponsive to standard resuscitation, especially those presenting with initial shockable rhythms like ventricular fibrillation (VF) or pulseless ventricular tachycardia (VT) [1–4]. Our previous research indicated the frequency of ECPR differs greatly between Osaka, Japan, and Singapore for refractory OHCA patients with initial shockable rhythm, and, among such patients, observed neurologically favorable outcomes and survival in Singapore was less than expected compared to Osaka [2]. However, this previous study had a substantial risk of selection bias, as the reference population in Osaka included only patients who were selected and transferred to tertiary care hospitals. To address concerns about selection bias and to confirm the robustness of previous results, we investigated the reproducibility and validity using population-based data. We also estimated the additional number of patients, using population-based data, who might obtain favorable outcomes if the resuscitation strategy in Osaka was implemented in Singapore. This study aimed to evaluate the validity of the previous results indicating outcome differences among OHCA patients with initial shockable rhythm in Singapore and Osaka using population-based data.

## Methods

This was a secondary analysis of two population-based OHCA databases in Singapore and Osaka, Japan, which were the All-Japan Utstein Registry and the Singapore Pan-Asian Resuscitation Outcomes Study (SG-PAROS) [5, 6]. Detailed information about these databases can be found in the Additional file 1 (S-Method 1). We extracted data on the Osaka Prefecture from this nationwide registry, with the aim of addressing the limitations of our previous study as mentioned in the background section. Similar to the previous study, [2] we included adult OHCA patients aged 18–74 with initial shockable rhythms, and excluded those without prehospital records, those who didn't receive resuscitation, had external causes or weren't in arrest when paramedics arrived. A machine-learning-based prediction model derived and validated as mentioned below using data from the Osaka data (derivation data 2010–2018, validation data 2019), was applied to the PAROS-SG data (2010–2019). The primary outcome of this study was one-month survival with favorable neurological outcomes defined as Cerebral Performance Category (CPC) 1 or 2. The secondary outcome of this study was one-month survival [7].

In the statistical analysis**,** patient characteristics, pre-hospital information, in-hospital procedures, and outcomes were described as continuous variables with median and interquartile range (IQR), while categorical variables were reported as numbers and percentages. In the main analysis, similar to the previous study, [2] we developed and validated machine-learning predictive models (random forest) of outcomes using the Osaka derivation data. The prediction models incorporated the following covariates: sex, age, witnessed events, bystander CPR, bystander automated external defibrillator, prehospital advanced airway management, prehospital adrenaline administration, and time from call to the hospital. We assessed the model's performance using the Osaka validation data. Then, we applied the prediction model to the SG-PAROS data in order to calculate the expected probability of outcomes. Subsequently, we computed the observed-expected ratio (OE ratio) and the observed-expected difference (OE difference) between the observed outcomes and the expected probability derived from the Osaka data with 95% confidence intervals (CI). Moreover, we estimated the incremental number of patients with the outcome by multiplying the OE difference by the number of cases. We calculated these values separately for patients with and without prehospital ROSC, to evaluate the potential impact of resuscitation strategies such as ECPR after hospital arrival but before ROSC. All statistical analyses were performed using R software, version 4.1.2 (R Foundation for Statistical Computing). The other details of the methods are described in the Additional file 1 (S-Method 2–4).

## Results

### Patient characteristics

For the Osaka Utstein Registry database, 3414 OHCA patients with an initial shockable rhythm were included in the analysis [the derivation cohort (*n* = 3,088) and the validation cohort (*n* = 326)]. Of the SG-PAROS database, 2905 patients with initial shockable rhythm were included in the analysis. The study flowchart is described in the Additional file 1 (S-Results 1). The median [IQR] age was 62 [51, 68] in the derivation cohort in Osaka, and 58 [50, 65] in the SG-PAROS. Good neurological outcomes were 32% (987/3,088) in Osaka and 17% (500/2,905) in Singapore. The prehospital ROSC were 36% (1,125/3,088) in Osaka and 27% (773/2,905) in Singapore, and among them, one-month good neurological outcomes were 70% (791/1,125) in Osaka and 57% (440/773), respectively. In comparison, among the patients without prehospital ROSC, good neurological outcomes were 10.0% (196/1,963) and 2.8% (60/2,132), respectively. The other details of characteristics, in-hospital information, and outcomes are described in Table 1 and the Additional file 1: (S-Results 2–5). ECMO was rarely performed in both populations of Singapore (with prehospital ROSC, 0.9% and without 0.5%). The prediction model derived from the Osaka derivation data performed well in the validation data. The details of the prediction model are described in the Additional file 1 (S-Results 6–7).

**Table 1:** Patient characteristics, in-hospital information, and outcomes

	Osaka 2010–2018 (Derivation cohort)			SG-PAROS 2010–2019		
Characteristic	Overall *n* = 3088	Prehospital ROSC, Yes *n* = 1125	Prehospital ROSC, No *n* = 1963	Overall *n* = 2905	Prehospital ROSC, Yes *n* = 773	Prehospital ROSC, No *n* = 2132
Male	2,576 (83%)	932 (83%)	1,644 (84%)	2,515 (87%)	668 (86%)	1,847 (87%)
Age (years)	62 (51, 68)	62 (51, 68)	62 (51, 68)	58 (50, 65)	57 (49, 64)	58 (50, 65)
Witness	2,453 (79%)	979 (87%)	1,474 (75%)	2,226 (77%)	618 (80%)	1,608 (75%)
Bystander CPR	1,491 (48%)	569 (51%)	922 (47%)	1,797 (62%)	528 (68%)	1,269 (60%)
Bystander AED	157 (5.1%)	68 (6.0%)	89 (4.5%)	369 (13%)	182 (24%)	187 (8.8%)
*Prehospital Airway*						
None	1,583 (51%)	732 (65%)	851 (43%)	390 (13%)	276 (36%)	114 (5.3%)
SGA	858 (28%)	229 (20%)	629 (32%)	2,498 (86%)	489 (63%)	2,009 (94%)
Intubation	647 (21%)	164 (15%)	483 (25%)	17 (0.6%)	8 (1.0%)	9 (0.4%)
Prehospital Drug	770 (25%)	215 (19%)	555 (28%)	2,072 (71%)	483 (62%)	1,589 (75%)
Time to ED arrival (min)	29 (24, 36)	29 (24, 36)	29 (24, 36)	36 (31, 41)	36 (31, 42)	35 (31, 41)
*In-hospital information*						
Admission	NA	NA	NA	1,118 (38%)	683 (88%)	435 (20%)
ECMO	NA	NA	NA	18 (0.6%)	7 (0.9%)	11 (0.5%)
PCI	NA	NA	NA	575 (20%)	379 (49%)	196 (9.2%)
TTM	NA	NA	NA	453 (16%)	282 (36%)	171 (8.0%)
One-month outcomes						
Good Neurological Outcome	987 (32%)	791 (70%)	196 (10.0%)	500 (17%)	440 (57%)	60 (2.8%)
Survival	1,294 (42%)	932 (83%)	362 (18%)	639 (22%)	524 (68%)	115 (5.4%)

Continuous variables are median and interquartile range, and categorical variables are number and percentage (%). *CPR* Cardiopulmonary resuscitation, *AED* Automated external defibrillator, *SGA* Supraglottic airway, *Prehospital drug* Prehospital adrenaline administration, *ROSC* Return of spontaneous circulation, *ED* Emergency department, *Shockable* Ventricular fibrillation and pulseless ventricular tachycardia, *Nonshockable* Pulseless electrical activity and asystole, *ECMO* Extracorporeal membrane oxygenation, *PCI* Percutaneous coronary intervention, *TTM* Targeted temperature management. *NA* Not applicable. In-hospital information in Osaka data is not available. The characteristics of the validation cohort are described in the Additional file 1

### The observed expected ratio, difference, and incremental number of patients

**Fig. 1:**
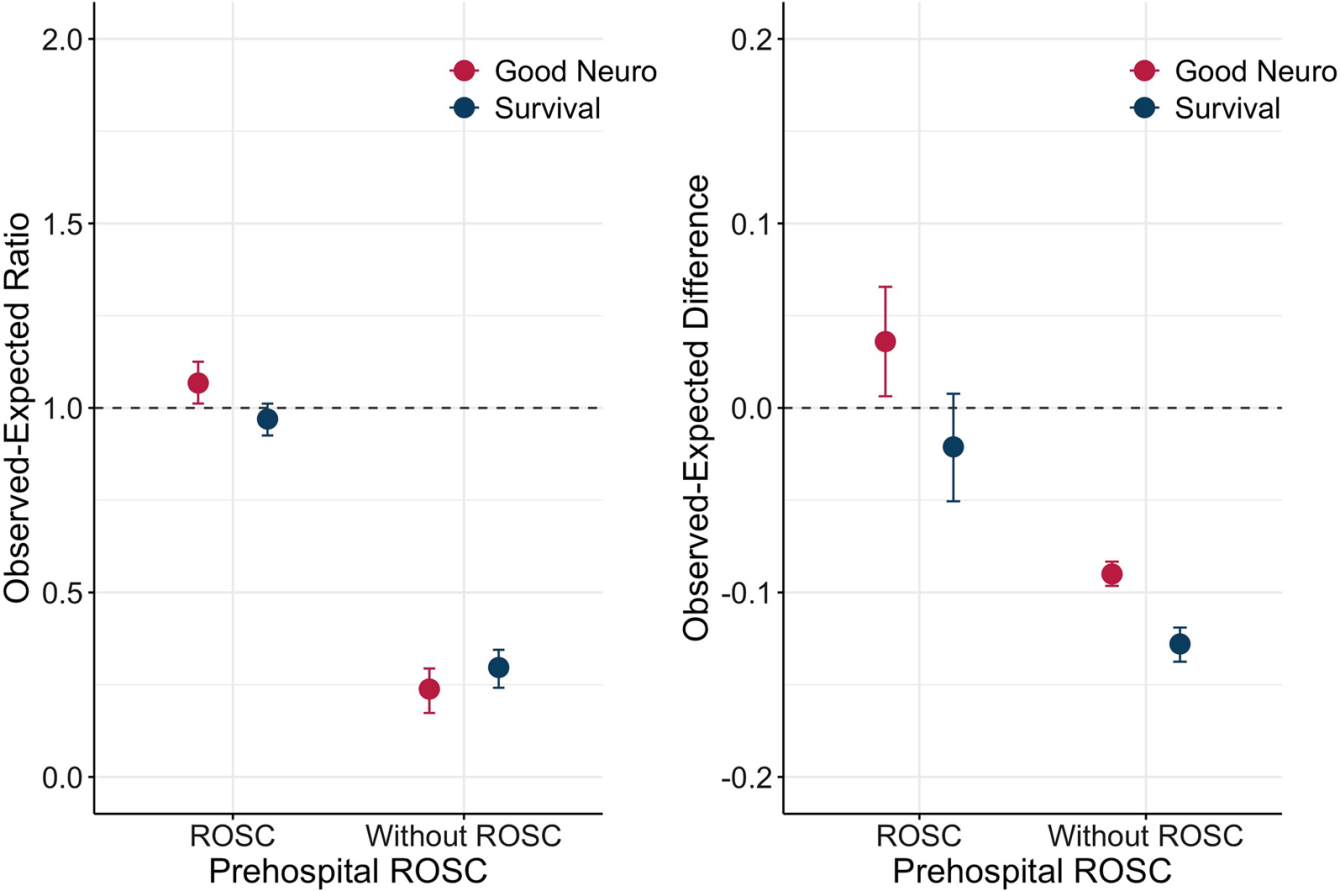
OE difference and the incremental number of patients with or without Prehospital ROSC. (Left) The Observed-Expected ratio and 95% confidence interval (CI). (Right) The Observed-Expected difference and 95% confidence interval (CI). *Good Neuro* One-month good neurological outcome, *Survival* One-month survival, ROSC, Return of spontaneous circulation

The one-month good neurological outcome in patients with prehospital ROSC in Singapore was the same or slightly better than expected compared to Osaka (OE ratio, 1.067 [95%CI 1.012 to 1.125] and OE difference, 0.036 [95%CI 0.006 to 0.066]). Conversely, the neurological outcome in patients without prehospital ROSC in Singapore was worse than expected compared to Osaka (OE ratio, 0.238 [95%CI 0.173 to 0.294] and OE difference, − 0.09 [95%CI − 0.096 to -0.083]), (Fig. 1). Similarly, for one-month survival, the result was consistent as follows: with prehospital ROSC, OE ratio, 0.97 [95%CI 0.925 to 1.012] and OE difference, − 0.021 [95%CI − 0.051 to 0.008], and without prehospital ROSC, OE ratio 0.297 [95%CI 0.242 to 0.345] and OE difference, -0.128 [95%CI -0.138 to -0.119], (Fig. 1).

The incremental one-month good neurological outcome in patients with prehospital ROSC was better than expected (27.8 [95% CI 4.9 to 50.7]), but was fewer than expected (− 191.9 [95% CI −205.6 to − 177.6]) in patients without prehospital ROSC) between 2010 and 2019. The incremental number of one-month survival cases had a similar trend to neurological outcomes (patients with pre-hospital ROSC: − 16.3 [95%CI − 39.1 to 5.9], patients without prehospital ROSC: − 272.7 [95%CI − 293.2 to − 253.8]). The incremental one-month good neurological outcome in each year is described in the Additional file 1 (S-Results 8).

## Discussion

This population-based observational study showed that the outcomes of OHCA patients without prehospital ROSC in Singapore were worse than expected derived from Osaka data even using population-based databases. These results were consistent with our previous research, mitigating previous selection bias concerns.

Similar to the previous study, we speculated that the outcome difference among patients without ROSC between Singapore and Osaka was related to the different availability of ECPR. First, outcomes were similar among patients with ROSC after adjusting the patients’ characteristics, which suggests the quality of post-resuscitation care after ROSC was comparable between Singapore and Osaka. In contrast, for patients without prehospital ROSC, ECMO was rarely performed (0.5%, 11/2,132) and hospital admission was considerably low (20%, 435/2,132) in Singapore while the percentage of ECPR cases was 30–60% and hospital admission was reported around 60–80% in the tertiary care hospitals in Osaka. [2] Considering the reports from RCTs that the hospital admission of the patients treated with ECPR were remarkably higher than the conventional group [e.g., INCEPTION trial, ECPR group 81% (57/70) vs 36% (23/64)], we suggested that the difference of hospital admission may be mainly caused by the difference of frequency of ECPR. [8, 9] In addition, the OE differences in neurological outcomes among patients without ROSC was approximately 9–10%, which is comparable to the effect magnitude of the ECPR strategy observed in a previous study in Japan (SAVE-J study) [12.3% (32/260) and 1.5% (3/194), with a risk difference of 10.8%], or the RCT in Prague, which also reported a risk difference of approximately 9.5%. [10, 11] Therefore, considering that the OE difference in our study closely aligns with the effect estimates from previous studies investigating aggressive ECPR strategies, we think it is reasonable to attribute the difference to ECPR strategies.

Several limitations should be acknowledged in this study, similar to the previous study. It didn't directly examine the link between ECPR policies and outcomes but instead highlighted potential differences in in-hospital care before ROSC between Osaka and Singapore. Outcomes might also be influenced by unmeasured factors like past medical history, frailty, and quality of resuscitation. A lack of detailed data limited our comparison of resuscitation specifics. Moreover, there are risks associated with the prediction model, such as overfitting and regularization bias. [12, 13].

## Conclusion

This study using two population-based databases indicated that the outcomes of OHCA patients without prehospital ROSC in Singapore were worse than expected compared to Osaka. The results are consistent with our previous research. We speculate that the outcome difference among patients without ROSC between Singapore and Osaka was due to different availability of ECPR.

(1455/1500 words).

### Supplementary Information

**Additional file 1. **Supplementary material.

## Data Availability

This study's datasets and/or analyses are not publicly available because the ethics committee did not permit it.
