# Supplementary material for: Comparing outcomes of out-of-hospital cardiac arrest patients with initial shockable rhythm in Singapore and Osaka using population-based databases
Source: Crit Care. 2023 Dec 6;27:479. doi: 10.1186/s13054-023-04771-5 (PMC10699037; doi:10.1186/s13054-023-04771-5)
Supplement: Supplementary file 1 — Additional file 1. Supplementary material. [file 13054_2023_4771_MOESM1_ESM.docx]

**aSupplementary file**

**Title:** Outcome Difference of Out-of-Hospital Cardiac Arrest Patients with Initial Shockable Rhythm Using Population-based Databases between Singapore and Osaka

**Contents**

| **S-Method 1** | **Description of databases and how to perform ECPR in Japan** |
| --- | --- |
| **S-Method 2** | **Description of the variables in the database** |
| **S-Method 3.** | **Missing imputation** |
| **S-Method 4.** | **Model derivation and validation** |
| **S-Results 1.** | **Study flowchart** |
| **S-Results 2.** | **Missing the data** |
| **S-Results 3.** | **The details of the characteristics in Osaka** |
| **S-Results 4.** | **Annual trend of patient characteristics** |
| **S-Results 5.** | **In-hospital care** |
| **S-Results 6.** | **Model features** |
| **S-Results 7.** | **Model performance in the validation cohort** |
| **S-Results 8.** | **The observed-expected difference** |

**S-Method 1. Description of databases and how to perform ECPR in Japan**

1. **The All-Japan Utstein Registry and the emergency medical system in OSAKA**

The All-Japan Utstein Registry is managed and compiled by the Fire and Disaster Management Agency (FDMA).(1-4) Fire departments nationwide collect data in accordance with the "Utstein Form Online Entry Procedure" and register the collected data in the Fire and Disaster Management Agency system. If it is clear that the error is caused by the system or conversion, correct the error if possible, or check with the respective fire department and correct it. (5) The data can be provided by the FDMA for research purposes.

Osaka Prefecture is an urban region in Japan, with an area of 1,905 km^2^, and it has a residential population of approximately 8.8 million in 2015.(6) In Osaka prefecture, 7,000-8,000 OHCA cases occur every year (5), and approximately 2,500 cases of them (25-30%) are transferred to the designated emergency and critical care centers (16 hospitals) certified by the Ministry of Health, Labor, and Welfare, and other cases were transferred to the other hospitals with emergency departments. Emergency and critical care centers are tertiary hospitals that provide advanced emergency care 24/7 basis to critically ill patients such as patients with an acute cardiovascular emergency, stroke, cardiac arrest, trauma, sepsis, and other conditions that cannot be treated in local hospitals. There is at least one per million population. The other hospitals with emergency departments also can provide advanced life support for cardiac arrest and some of them can provide intensive care or percutaneous coronary intervention, but in most of them, the capability to provide the ECPR is limited. In Japan, certified paramedics can provide advanced airway management and administer adrenaline in prehospital settings under the online supervision of designated emergency physicians. However, for OHCA patients with a shockable rhythm, the local protocol in the Tokyo region restricts prehospital adrenaline administration to reduce the time to initiate ECPR.(7) Similarly, in other urban areas like Osaka, there is a tendency to limit prehospital administration and advanced airway under the supervision of medical control doctors.

1. **SG-PAROS database**

Singapore, situated in Southeast Asia just north of the equator, is a small island city-state renowned for its modern infrastructure, high population density, and diverse populace. With an approximate population of 5.7 million people, Singapore covers a total land area of around 719.1 km^2^. The Pan-Asian Resuscitation Outcomes Study (PAROS) is an extensive registry that gathers data on out-of-hospital cardiac arrests (OHCAs) occurring in the Asia-Pacific region. Established in 2009, PAROS encompasses OHCAs treated by emergency medical services (EMS) or presented at emergency departments (EDs). The data collected by PAROS adheres to the Utstein-style guidelines, ensuring a standardized reporting method for OHCAs. This includes details on prehospital care, medical procedures administered, and outcomes of the OHCAs. The primary aim of PAROS is to enhance the understanding of OHCAs in the Asia-Pacific region and identify strategies for improving outcomes. Notably, 99% of the cardiac arrest patients in this database were transferred to Singapore's eight tertiary care hospitals. (8) In this database, 99% of the cardiac arrest patients were transferred to the 8 tertiary care hospitals in Singapore.

1. **How to perform ECPR in Japan**

In Japan, the initial significant study on extracorporeal cardiopulmonary resuscitation (ECPR), known as the SAVE-J study, was a large-scale prospective observational study conducted between 2008 and 2011, involving 46 institutions. (9, 10) This study provided evidence of the potentially beneficial effects of ECPR. Since then, the eligibility criteria outlined in the SAVE-J study, including initial shockable rhythm, time from call to hospital arrival within 45 minutes, and age younger than 75 years old, have been widely regarded as important references in Japan. (9, 10) However, it is important to note that formal, universally defined criteria for ECPR implementation are lacking in nearly half of the tertiary care hospitals in Japan. Instead, the decision regarding ECPR candidacy is typically made by the attending physician responsible for the patient's care in these hospitals. (11, 12) Typically, a standby call from the ambulance alerts the staff, including the emergency physician, cardiologists, perfusionist, and nurses, who then assemble in the emergency department or catheter suite. Approximately half of the hospitals perform ECPR in the catheter suite, while others conduct it in the emergency departments, or emergency departments equipped with angiography facilities. (12) Vascular access is commonly obtained using percutaneous access guided by ultrasonography, and the cannulation procedure is typically performed by emergency physicians and/or cardiologists. (12)

During the ECPR procedure, defibrillation is generally limited to persistent shockable rhythms if it was not administered during prehospital settings, and sometimes drug administration is also omitted. The median time from the patient's arrival at the hospital to ECMO initiation is approximately 15-30 minutes, with an interquartile range of 15-32 minutes. (11, 12) Although some hospitals are conducting prehospital ECPR experiments using physician-staffed ambulances, the number of actual cases remains limited.

**S-Method 2. Description of the variables in the database**

| Variable | Description |
| --- | --- |
| Basic demographics | |
| Sex | Sex (Male/Female) |
| Age | Age (years) |
| Prehospital information (Utstein style) | |
| Witnessed | Witness of collapse (Yes/No) Not registered is categorized as No. |
| Bystander CPR | Bystander CPR (Yes/ No) Not registered is categorized as No. |
| Initial cardiac rhythm | The cardiac rhythm initially confirmed by paramedics at the scene |
| Bystander AED | Defibrillation performed by Bystander (Yes/No) Not registered is categorized as No. |
| Prehospital advanced airway management | Advanced airway management performed by paramedics (Intubation/Supraglottic airway/None) Not registered is categorized as No. |
| IV adrenaline | Administrations of adrenaline via iv route by paramedics (Yes/ No) Not registered is categorized as No. |
| Prehospital ROSC | ROSC at the scene or during the transportation (Yes/ No) Not registered is categorized as No. |
| Time from Call to Hospital | The time from emergency call to the patients arrived at hospital (minute) |
| In-hospital information | |
| Disposition in the ED | Death in ED without admission, or admission to the hospital. The transfer to another hospital was categorized as admission to the hospital. |
| ECMO | ECMO was performed (Yes/No) Not registered is categorized as No. |
| PCI | The emergency PCI was performed (Yes/No) Not registered is categorized as No. |
| TTM | TTM was performed (Yes/No) Not registered is categorized as No. |
| Survival | The status at the 30th-day post-arrest or the time of discharge. Survival: The patient was discharged alive or remains in the hospital on the 30th day post-arrest. Dead: Died in hospital. |
| Neurological outcome | The status at the 30th-day post-arrest or the time of discharge. Good: Survival with favorable neurological outcomes defined as CPC 1 or 2, Poor: the status CPC3or 4, or death (CPC5) |

CPR, Cardiopulmonary resuscitation, VF, Ventricular fibrillation, VT, Ventricular tachycardia, PEA: Pulseless electrical activity, ROSC, Return of spontaneous circulation, AED, Automated external defibrillator, ECMO, Extracorporeal membrane oxygenation, PCI, Percutaneous coronary intervention, TTM, Targeted temperature management. CPC, Cerebral Performance Category.

**S-Method 3. Missing imputation**

We treated extreme outliers or contradictory data as missing. To address missing variables, we utilized the "missForest" package, a machine learning-based imputation technique, to impute the missing values. (13, 14) This imputation technique is a nonparametric algorithm that can accommodate nonlinearities and interactions, and the single point estimates can be generated accurately by a random forest model. (13, 14) The use of a random forest model has the advantage of being able to handle both continuous and categorical responses, requiring minimal tuning, and providing an internally cross-validated error estimate. This imputation technique has been shown to be reliable and valid compared to other imputation methods such as k-nearest neighbors’ imputation or multivariate imputation using chained equations. (13, 14) Missingness was imputed using all predictors and outcomes.

**S-Method 4. Model derivation and validation**

**Derivation and validation cohort**

We divided the included patients in into two groups: a derivation cohort from 2010-2018 and a validation cohort from 2019. The purpose of this division was to validate the prediction model by confirming its generalizability to a slightly different patient population. Generally, external validation of the prediction model requires a different patient spectrum. (15, 16) We chose to divide the data chronologically because they wanted to predict the outcome if OHCA patients in other areas were transferred to hospitals in Osaka and received the average treatment as a reference.

**Model derivation**

Based on previous studies, we chose the random forest model in the main analysis which is one of the most common machine-learning models. (17-20) Random forest is an ensemble learning method that consists of hundreds or thousands of decision trees. (21) It trains each one on a slightly different set of observations using bootstrapping, and the final predictions are made by averaging the predictions of each individual tree. For developing the random forest, we performed optimization of the hyperparameters by grid search strategy using the “ranger” and “caret” packages. (22, 23) To understand the contribution of predictors to the models, we showed that the variable importance scaled as the maximum value is 100.(23, 24)

The prediction models were derived to incorporate the following covariates: sex, age, witnessed events, bystander CPR, bystander AED, prehospital advanced airway management, prehospital adrenaline administration, and time from call to the hospital. These covariates were selected based on their availability in both datasets, their clinical importance, and their ability to adjust for differences in case mix upon hospital arrival, thereby allowing evaluation of potential differences in in-hospital treatment strategies between regions.

**Model evaluation in the validation cohort**

For the assessment of predictive performance, developed models were applied to the validation cohort as external validation. The receiver operating curves (ROCs) were drawn, and the area under the curve (AUC) with the 95% confidence interval (95% CI) were calculated as discrimination. The model’s performance was also evaluated based on the C index, the Nagelkerke R^2^ value, calibration intercept and slope, and the Brier score. (15) Calibration plots were also created to graphically indicate the association between the predicted and observed outcome using tertile.

**Prediction model application using the SG-PAROS data**

We computed the observed-expected ratio (OE ratio) and difference (OE difference) between the observed outcomes and the expected probability derived from the Osaka data. Moreover, we estimated the incremental number of patients with the outcome by multiplying the OE difference by the number of cases.) For instance, if there were 10 cases with a mean expected probability of survival at 0.50 (50%), and only 2 of them survived (20%), the OE difference would be -0.3 (= 0.2 - 0.5), indicating that the observed outcome is worse than expected by 30%. The 95% confidence interval (CI) was calculated using the bootstrapping procedure (1000 times). Further, if the OE difference is -0.3 among a group of ten patients, the incremental number of patients would be -3.0, indicating 3.0 fewer patients with the outcome than expected.

**S-Results 1. Study flowchart**

OHCA, out-of-hospital cardiac arrest, ROSC, Return of spontaneous circulation.

**S-Results 2. Missing the data**

| Characteristic | Osaka data  (n = 3,414) | SG-PAROS data  (n = 2,905) |
| --- | --- | --- |
| Sex | 0 (0%) | 0 (0%) |
| Age | 0 (0%) | 0 (0%) |
| Witnessed | 0 (0%) | 0 (0%) |
| Bystander CPR | 0 (0%) | 0 (0%) |
| Bystander AED | 0 (0%) | 0 (0%) |
| Prehospital Airway | 0 (0%) | 357(12.3%) |
| Prehospital Drug | 0 (0%) | 0 (0%) |
| Prehospital ROSC | 0 (0%) | 0 (0%) |
| Time to ED arrival | 0 (0%) | 16 (0.6%) |
| Outcome |  |  |
| 30-day Survival | 0 (0%) | 2 (0.1%) |
| 30-day Neurological outcome | 0 (0%) | 2 (0.1%) |

Number and percentage (%). CPR, Cardiopulmonary resuscitation, ROSC, Return of spontaneous circulation, AED, Automated external defibrillator, ROSC, Return of spontaneous circulation, ED: Emergency department.

**S-Results 3. The details of the Characteristics in Osaka**

| Characteristic | Derivation Cohort |  | Validation Cohort |  |
| --- | --- | --- | --- | --- |
|  | n = 3,088 | Overall, n = 326 | Prehospital ROSC, Yes, n = 125 | Prehospital ROSC, No,  n = 201 |
| Male | 2,576 (83%) | 284 (87%) | 107 (86%) | 177 (88%) |
| Age (years) | 62 (51, 68) | 60 (50, 69) | 58 (47, 68) | 60 (52, 69) |
| Witness | 2,453 (79%) | 255 (78%) | 106 (85%) | 149 (74%) |
| Bystander CPR | 1,491 (48%) | 115 (35%) | 43 (34%) | 72 (36%) |
| Bystander AED | 157 (5.1%) | 24 (7.4%) | 11 (8.8%) | 13 (6.5%) |
| Prehospital Airway |  |  |  |  |
| None | 1,583 (51%) | 195 (60%) | 94 (75%) | 101 (50%) |
| SGA | 858 (28%) | 79 (24%) | 15 (12%) | 64 (32%) |
| Intubation | 647 (21%) | 52 (16%) | 16 (13%) | 36 (18%) |
| Prehospital Drug | 770 (25%) | 96 (29%) | 26 (21%) | 70 (35%) |
| Prehospital ROSC | 1,125 (36%) | 125 (38%) | 125 (100%) | 0 (0%) |
| Time to ED arrival (min) | 29 (24, 36) | 29 (23, 35) | 29 (23, 33) | 29 (23, 35) |
| Good Neurological Outcome | 987 (32%) | 110 (34%) | 84 (67%) | 26 (13%) |
| Survival | 1,294 (42%) | 148 (45%) | 95 (76%) | 53 (26%) |
| Year |  |  |  |  |
| 2010 | 353 (11%) | 0 (0%) | 0 (0%) | 0 (0%) |
| 2011 | 314 (10%) | 0 (0%) | 0 (0%) | 0 (0%) |
| 2012 | 358 (12%) | 0 (0%) | 0 (0%) | 0 (0%) |
| 2013 | 214 (6.9%) | 0 (0%) | 0 (0%) | 0 (0%) |
| 2014 | 350 (11%) | 0 (0%) | 0 (0%) | 0 (0%) |
| 2015 | 368 (12%) | 0 (0%) | 0 (0%) | 0 (0%) |
| 2016 | 392 (13%) | 0 (0%) | 0 (0%) | 0 (0%) |
| 2017 | 374 (12%) | 0 (0%) | 0 (0%) | 0 (0%) |
| 2018 | 365 (12%) | 0 (0%) | 0 (0%) | 0 (0%) |
| 2019 | 0 (0%) | 326 (100%) | 125 (100%) | 201(100%) |

Continuous variables are median and interquartile range, and categorical variables are number and percentage (%). CPR, Cardiopulmonary resuscitation, AED, Automated external defibrillator, SGA, Supraglottic airway, Prehospital drug, prehospital adrenaline administration, ROSC, Return of spontaneous circulation, ED, Emergency department, Shockable: Ventricular fibrillation and pulseless ventricular tachycardia, ECMO, Extracorporeal membrane oxygenation, PCI, Percutaneous coronary intervention, TTM, Targeted temperature management. In-hospital information in Osaka data is not available.

**S-Results 4. Annual trend of patient characteristics**

**Patient characteristics by year (Osaka, derivation cohort)**

| Characteristic | 2010,  N = 353 | 2011,  N = 314 | 2012,  N = 358 | 2013,  N = 214 | 2014,  N = 350 | 2015,  N = 368 | 2016,  N = 392 | 2017,  N = 374 | 2018,  N = 365 |
| --- | --- | --- | --- | --- | --- | --- | --- | --- | --- |
| Male | 300 (85%) | 261 (83%) | 297 (83%) | 169 (79%) | 293 (84%) | 304 (83%) | 322 (82%) | 319 (85%) | 311 (85%) |
| Age (years) | 61 (51, 67) | 62 (52, 67) | 63 (54, 69) | 61 (51, 69) | 64 (53, 70) | 60 (48, 68) | 62 (50, 68) | 63 (53, 69) | 60 (50, 68) |
| Witness | 281 (80%) | 237 (75%) | 275 (77%) | 161 (75%) | 266 (76%) | 298 (81%) | 325 (83%) | 307 (82%) | 303 (83%) |
| Bystander CPR | 163 (46%) | 124 (39%) | 151 (42%) | 112 (52%) | 190 (54%) | 198 (54%) | 210 (54%) | 190 (51%) | 153 (42%) |
| Bystander AED | 11 (3.1%) | 10 (3.2%) | 11 (3.1%) | 9 (4.2%) | 24 (6.9%) | 15 (4.1%) | 21 (5.4%) | 20 (5.3%) | 36 (9.9%) |
| Prehospital Airway |  |  |  |  |  |  |  |  |  |
| None | 143 (41%) | 138 (44%) | 183 (51%) | 141 (66%) | 187 (53%) | 194 (53%) | 206 (53%) | 192 (51%) | 199 (55%) |
| SGA | 129 (37%) | 115 (37%) | 100 (28%) | 27 (13%) | 87 (25%) | 103 (28%) | 102 (26%) | 98 (26%) | 97 (27%) |
| Intubation | 81 (23%) | 61 (19%) | 75 (21%) | 46 (21%) | 76 (22%) | 71 (19%) | 84 (21%) | 84 (22%) | 69 (19%) |
| Prehospital Drug | 77 (22%) | 77 (25%) | 83 (23%) | 37 (17%) | 90 (26%) | 83 (23%) | 104 (27%) | 109 (29%) | 110 (30%) |
| Prehospital ROSC | 122 (35%) | 111 (35%) | 134 (37%) | 78 (36%) | 103 (29%) | 150 (41%) | 143 (36%) | 130 (35%) | 154 (42%) |
| Time to ED arrival (min) | 28 (23, 33) | 28 (24, 34) | 28 (24, 35) | 28 (22, 36) | 29 (25, 35) | 29 (24, 35) | 35 (26, 82) | 29 (24, 36) | 30 (25, 36) |
| Good Neurological Outcome | 93 (26%) | 86 (27%) | 104 (29%) | 65 (30%) | 100 (29%) | 142 (39%) | 145 (37%) | 122 (33%) | 130 (36%) |
| Survival | 141 (40%) | 120 (38%) | 138 (39%) | 85 (40%) | 128 (37%) | 170 (46%) | 176 (45%) | 159 (43%) | 177 (48%) |

Continuous variables are median and interquartile range, and categorical variables are number and percentage (%). CPR, Cardiopulmonary resuscitation, AED, Automated external defibrillator, SGA, Supraglottic airway, Prehospital drug, prehospital adrenaline administration, ROSC, Return of spontaneous circulation, ED, Emergency department.

**Patient characteristics by year (Singapore)**

| Characteristic | 2010,  N = 169 | 2011,  N = 213 | 2012,  N = 241 | 2013,  N = 253 | 2014,  N = 282 | 2015,  N = 308 | 2016,  N = 323 | 2017,  N = 348 | 2018,  N = 364 | 2019,  N = 404 |
| --- | --- | --- | --- | --- | --- | --- | --- | --- | --- | --- |
| Male | 149 (88%) | 186 (87%) | 199 (83%) | 224 (89%) | 245 (87%) | 267 (87%) | 276 (85%) | 301 (86%) | 319 (88%) | 349 (86%) |
| Age (years) | 58 (50, 64) | 60 (52, 66) | 58 (51, 65) | 57 (50, 65) | 56 (49, 63) | 58 (49, 65) | 56 (49, 64) | 58 (50, 65) | 58 (51, 64) | 59 (51, 65) |
| Witness | 85 (50%) | 174 (82%) | 172 (71%) | 207 (82%) | 226 (80%) | 246 (80%) | 263 (81%) | 280 (80%) | 284 (78%) | 289 (72%) |
| Bystander CPR | 56 (33%) | 80 (38%) | 105 (44%) | 139 (55%) | 182 (65%) | 204 (66%) | 222 (69%) | 260 (75%) | 259 (71%) | 290 (72%) |
| Bystander AED | 9 (5.3%) | 8 (3.8%) | 9 (3.7%) | 23 (9.1%) | 23 (8.2%) | 29 (9.4%) | 42 (13%) | 75 (22%) | 64 (18%) | 87 (22%) |
| Prehospital Airway |  |  |  |  |  |  |  |  |  |  |
| None | 28 (17%) | 22 (10%) | 21 (8.7%) | 25 (9.9%) | 38 (13%) | 40 (13%) | 47 (15%) | 53 (15%) | 47 (13%) | 69 (17%) |
| SGA | 139 (82%) | 188 (88%) | 219 (91%) | 228 (90%) | 242 (86%) | 266 (86%) | 275 (85%) | 291 (84%) | 316 (87%) | 334 (83%) |
| Intubation | 2 (1.2%) | 3 (1.4%) | 1 (0.4%) | 0 (0%) | 2 (0.7%) | 2 (0.6%) | 1 (0.3%) | 4 (1.1%) | 1 (0.3%) | 1 (0.2%) |
| Prehospital Drug | 99 (59%) | 104 (49%) | 140 (58%) | 161 (64%) | 173 (61%) | 195 (63%) | 200 (62%) | 232 (67%) | 364 (100%) | 404 (100%) |
| Prehospital ROSC | 25 (15%) | 29 (14%) | 34 (14%) | 40 (16%) | 51 (18%) | 75 (24%) | 111 (34%) | 113 (32%) | 137 (38%) | 158 (39%) |
| Time to ED arrival (min) | 32 (28, 37) | 32 (27, 36) | 32 (28, 38) | 34 (30, 41) | 36 (32, 41) | 36 (31, 41) | 37 (33, 42) | 38 (33, 44) | 37 (32, 42) | 37 (32, 43) |
| Good Neurological Outcome | 13 (7.7%) | 20 (9.4%) | 21 (8.7%) | 27 (11%) | 39 (14%) | 51 (17%) | 73 (23%) | 64 (18%) | 83 (23%) | 109 (27%) |
| Survival | 19 (11%) | 32 (15%) | 31 (13%) | 42 (17%) | 52 (18%) | 65 (21%) | 94 (29%) | 77 (22%) | 104 (29%) | 123 (30%) |

Continuous variables are median and interquartile range, and categorical variables are number and percentage (%). CPR, Cardiopulmonary resuscitation, AED, Automated external defibrillator, SGA, Supraglottic airway, Prehospital drug, prehospital adrenaline administration, ROSC, Return of spontaneous circulation, ED, Emergency department. After 2015, several measures like dispatcher-assisted bystander CPR program, smartphone applications to activate volunteers, etc. have been implemented in Singapore, subsequently, the bystander CPR and AED applications have increased. (25)

**S-Results 5. In-hospital care in Singapore**

**Patients with ROSC**

**
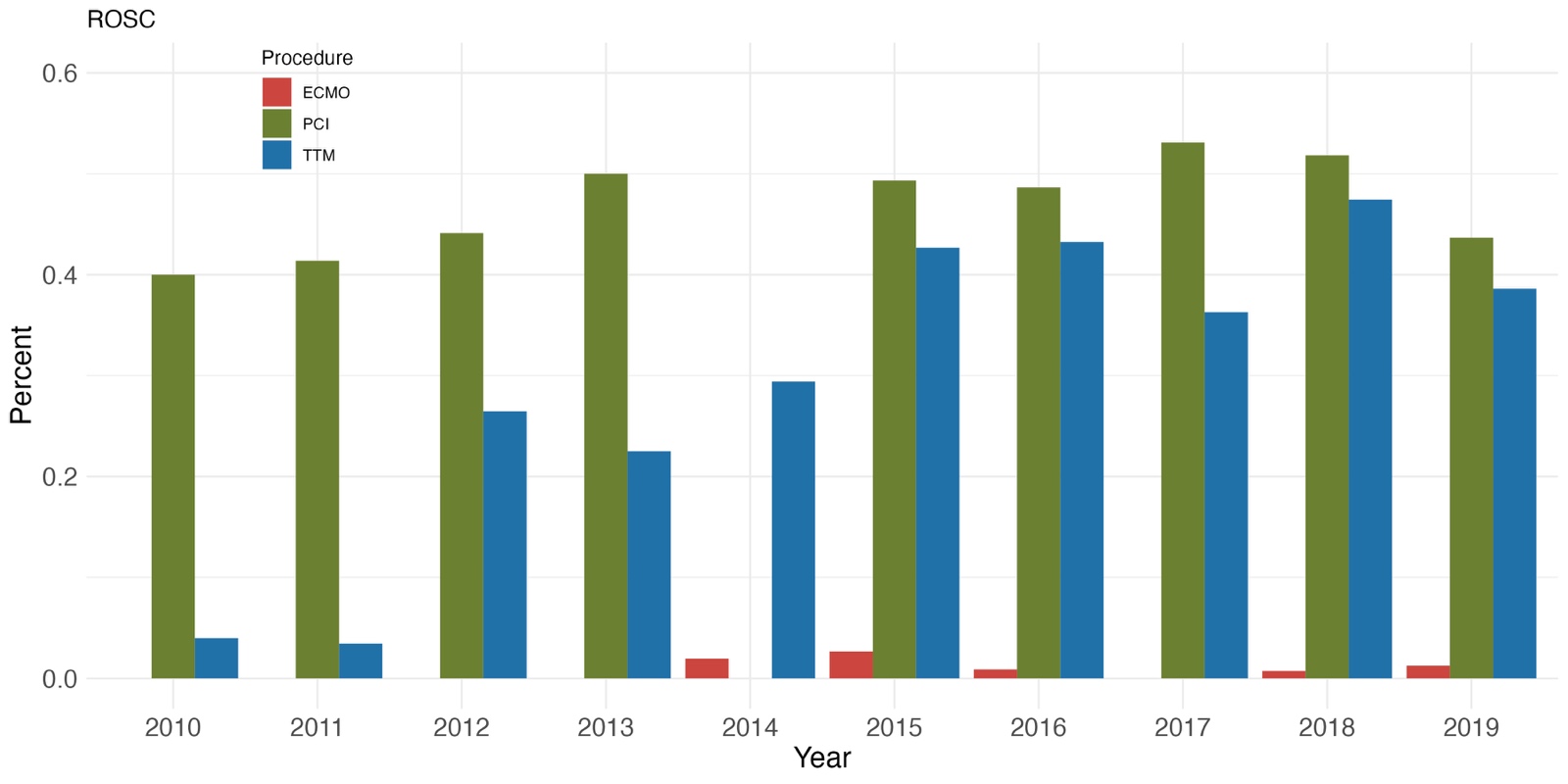
**

**Patients without ROSC**

**
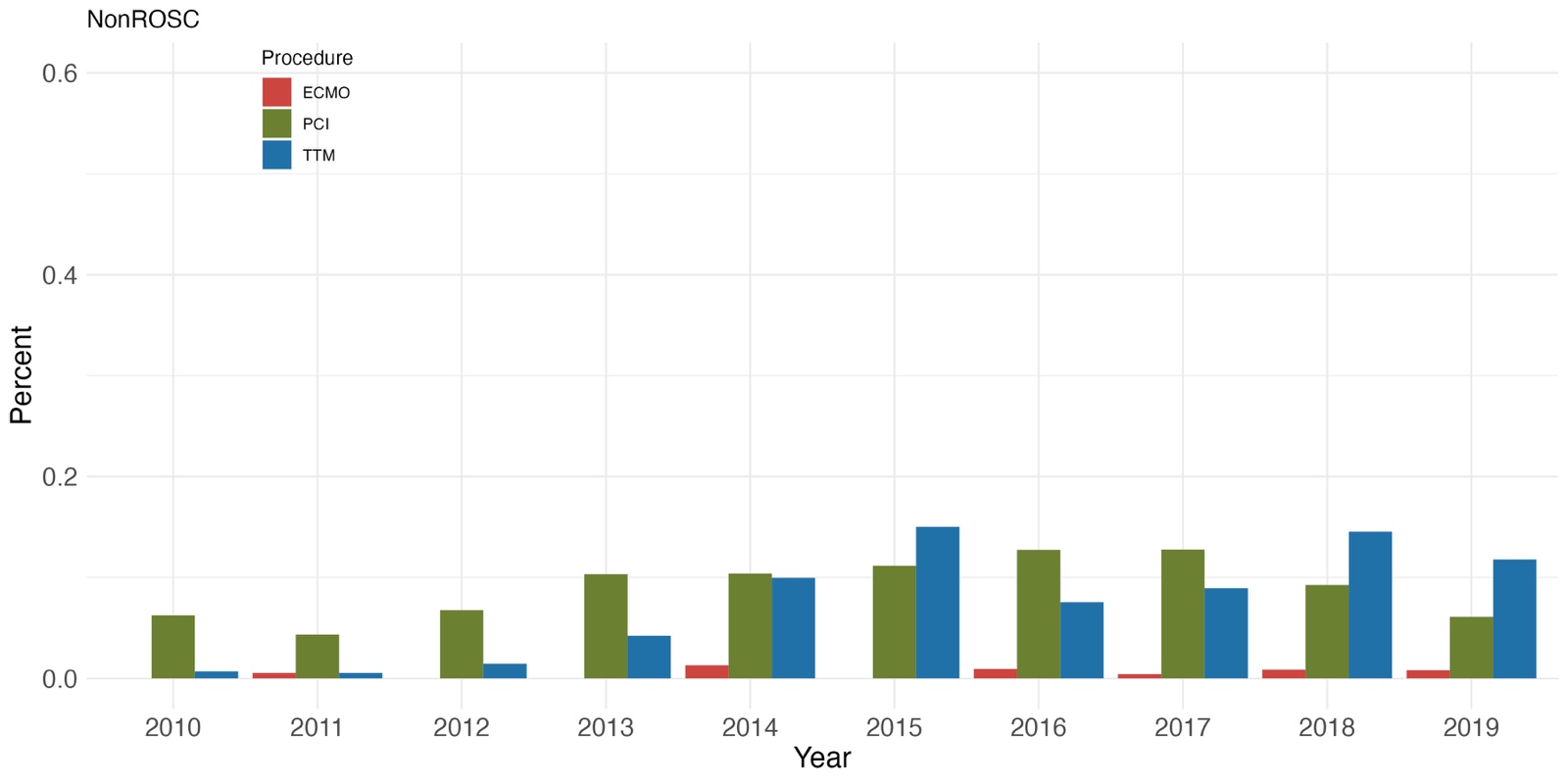
**

ROSC, return of spontaneous circulation, ECMO, Extracorporeal membrane oxygenation, PCI, Percutaneous coronary intervention, TTM, Targeted temperature management.

**S-Results 6. Model features**

**Random Forest**

| Predictors | Neurological outcome | Survival |
| --- | --- | --- |
| Sex (Female) | 0 | 0 |
| Age | 1.9 | 2.2 |
| Witness (Yes) | 1.8 | 2.3 |
| Bystander CPR (Yes) | 0.2 | 0 |
| Bystander AED (Yes) | 0.2 | 0.7 |
| Prehospital Airway (None) | 9.5 | 7.7 |
| Prehospital Airway (SGA) | 2 | 2.2 |
| Prehospital Drug (Yes) | 6 | 3 |
| Prehospital ROSC (Yes) | 100 | 100 |
| Time to ED arrival | 0.3 | 4.2 |

CPR, Cardiopulmonary resuscitation, AED, Automated external defibrillator, SGA, Supraglottic airway, Prehospital drug, prehospital adrenaline administration, ROSC, Return of spontaneous circulation, ED, Emergency department,

**S-Results 7. Model performance in the validation cohort**

**Random Forest**

| **Outcome** | **AUC** | **R^2^** | **Brier** | **Intercept** | **Slope** |
| --- | --- | --- | --- | --- | --- |
| Good Neurological Outcome | 0.861 | 0.472 | 0.134 | 0.16 | 1.266 |
| Survival | 0.804 | 0.357 | 0.177 | 0.115 | 0.959 |

**Receiver operating curve (ROC)**


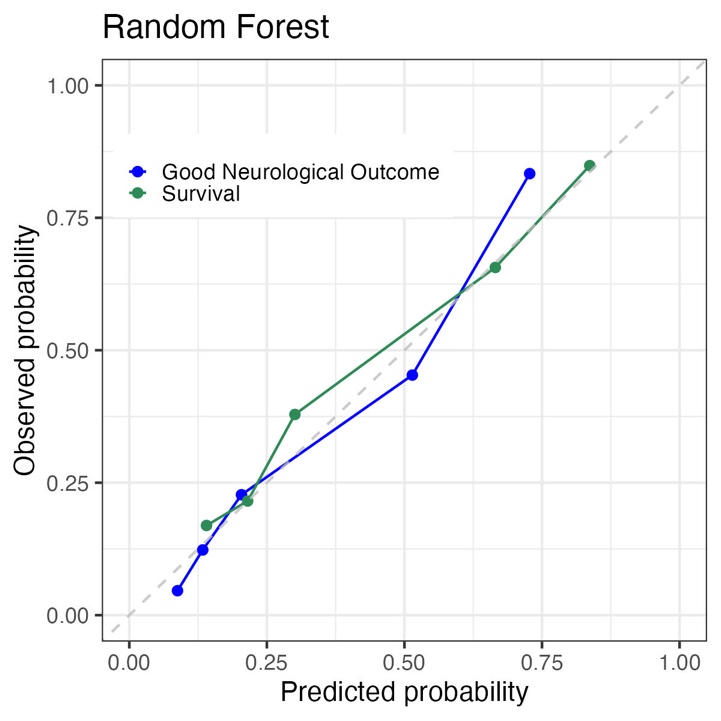
Calibration Plot

Diagonal lines imply the perfect agreement between prediction and observation.

**S-Results 8. The observed-expected difference**

Among patients with prehospital ROSC

| Year | OE difference [95%CI] | | n | Incremental N of cases [95%CI] | |
| --- | --- | --- | --- | --- | --- |
|  | Neuro outcome | Survival |  | Neuro outcome | Survival |
| 2010 | -0.113 [-0.294 to 0.073] | -0.153 [-0.333 to 0.013] | 25 | -2.8 [-7.3 to 1.8] | -3.8 [-8.3 to 0.3] |
| 2011 | 0.067 [-0.085 to 0.222] | 0.075 [-0.054 to 0.225] | 29 | 1.9 [-2.5 to 6.4] | 2.2 [-1.6 to 6.5] |
| 2012 | -0.018 [-0.150 to 0.101] | -0.098 [-0.235 to 0.049] | 34 | -0.6 [-5.1 to 3.4] | -3.3 [-8.0 to 1.7] |
| 2013 | -0.062 [-0.199 to 0.083] | -0.038 [-0.161 to 0.094] | 40 | -2.5 [-7.9 to 3.3] | -1.5 [-6.4 to 3.8] |
| 2014 | 0.047 [-0.064 to 0.159] | 0.02 [-0.079 to 0.121] | 51 | 2.4 [-3.3 to 8.1] | 1.0 [-4.0 to 6.1] |
| 2015 | -0.044 [-0.145 to 0.048] | -0.071 [-0.17 to 0.031] | 75 | -3.3 [-10.8 to 3.6] | -5.3 [-12.7 to 2.3] |
| 2016 | -0.009 [-0.087 to 0.065] | -0.051 [-0.134 to 0.027] | 111 | -1.0 [-9.7 to 7.2] | -5.6 [-14.9 to 3.0] |
| 2017 | -0.078 [-0.157 to 0.004] | -0.134 [-0.218 to -0.048] | 113 | -8.8 [-17.8 to 0.4] | -15.1 [-24.7 to -5.4] |
| 2018 | 0.101 [0.021 to 0.182] | 0.033 [-0.047 to 0.110] | 137 | 13.8 [2.9 to 24.9] | 4.6 [-6.5 to 15.1] |
| 2019 | 0.182 [0.115 to 0.252] | 0.068 [0.002 to 0.139] | 158 | 28.7 [18.2 to 39.8] | 10.7 [0.3 to 22.0] |

Among patients without prehospital ROSC

| Year | OE difference [95%CI] | | n | Incremental N of case [95%CI] | |
| --- | --- | --- | --- | --- | --- |
|  | Neuro outcome | Survival |  | Neuro outcome | Survival |
| 2010 | -0.083 [-0.107 to -0.063] | -0.137 [-0.174 to -0.106] | 25 | -12.0 [-15.4 to -9.1] | -19.8 [-25.0 to -15.3] |
| 2011 | -0.109 [-0.127 to -0.096] | -0.149 [-0.185 to -0.121] | 29 | -20.0 [-23.3 to -17.7] | -27.5 [-34.1 to -22.3] |
| 2012 | -0.103 [-0.122 to -0.088] | -0.147 [-0.178 to -0.118] | 34 | -21.4 [-25.4 to -18.2] | -30.4 [-36.9 to -24.4] |
| 2013 | -0.093 [-0.120 to -0.073] | -0.125 [-0.162 to -0.095] | 40 | -19.8 [-25.6 to -15.5] | -26.6 [-34.6 to -20.2] |
| 2014 | -0.098 [-0.120 to -0.080] | -0.128 [-0.159 to -0.099] | 51 | -22.5 [-27.7 to -18.5] | -29.5 [-36.8 to -22.8] |
| 2015 | -0.074 [-0.105 to -0.047] | -0.115 [-0.152 to -0.085] | 75 | -17.3 [-24.5 to -11.0] | -26.9 [-35.4 to -19.8] |
| 2016 | -0.073 [-0.104 to -0.045] | -0.087 [-0.131 to -0.053] | 111 | -15.5 [-22.1 to -9.5] | -18.5 [-27.8 to -11.2] |
| 2017 | -0.094 [-0.119 to -0.073] | -0.134 [-0.162 to -0.107] | 113 | -22.1 [-28.1 to -17.2] | -31.4 [-38.0 to -25.2] |
| 2018 | -0.092 [-0.114 to -0.076] | -0.132 [-0.159 to -0.109] | 137 | -20.9 [-25.8 to -17.1] | -30.0 [-36.0 to -24.7] |
| 2019 | -0.083 [-0.105 to -0.066] | -0.131 [-0.156 to -0.111] | 158 | -20.3 [-25.9 to -16.1] | -32.2 [-38.5 to -27.4] |

Neuro outcome: One-month good neurological outcome

**Reference**

1. Kitamura T, Iwami T, Kawamura T, Nagao K, Tanaka H, Hiraide A. Nationwide public-access defibrillation in Japan. N Engl J Med. 362. United States: 2010 Massachusetts Medical Society; 2010. p. 994-1004.

2. Kitamura T, Kiyohara K, Sakai T, Matsuyama T, Hatakeyama T, Shimamoto T, et al. Public-Access Defibrillation and Out-of-Hospital Cardiac Arrest in Japan. N Engl J Med. 2016;375(17):1649-59.

3. Jacobs I, Nadkarni V, Bahr J, Berg RA, Billi JE, Bossaert L, et al. Cardiac arrest and cardiopulmonary resuscitation outcome reports: update and simplification of the Utstein templates for resuscitation registries: a statement for healthcare professionals from a task force of the International Liaison Committee on Resuscitation (American Heart Association, European Resuscitation Council, Australian Resuscitation Council, New Zealand Resuscitation Council, Heart and Stroke Foundation of Canada, InterAmerican Heart Foundation, Resuscitation Councils of Southern Africa). Circulation. 110. United States2004. p. 3385-97.

4. Yamada T, Kitamura T, Hayakawa K, Yoshiya K, Irisawa T, Abe Y, et al. Rationale, design, and profile of Comprehensive Registry of In-Hospital Intensive Care for OHCA Survival (CRITICAL) study in Osaka, Japan. J Intensive Care. 2016;4:10.

5. Fire and Disaster Management Agency of the Ministry of Internal Affairs and Communications  website [Available from: http://www.fdma.go.jp/neuter/topics/fieldList9_3.html.

6. Communications MoIAa. Statistics Bureau [Available from: https://www.stat.go.jp/english/.

7. Shibahashi K. Impact of a revised prehospital care protocol that restricts prehospital defibrillation attempts and omits adrenaline administration on the prehospital care and the outcomes of patients experiencing out–of–hospital cardiac arrest with initial shockable cardiac rhythm: an interrupted time–series analysis. Nihon Kyukyu Igakukai Zasshi: Journal of Japanese Association for Acute Medicine. 2021;32(8):399-407.

8. Ong ME, Shin SD, Tanaka H, Ma MH, Khruekarnchana P, Hisamuddin N, et al. Pan-Asian Resuscitation Outcomes Study (PAROS): rationale, methodology, and implementation. Acad Emerg Med. 2011;18(8):890-7.

9. Sakamoto T, Morimura N, Nagao K, Asai Y, Yokota H, Nara S, et al. Extracorporeal cardiopulmonary resuscitation versus conventional cardiopulmonary resuscitation in adults with out-of-hospital cardiac arrest: a prospective observational study. Resuscitation. 2014;85(6):762-8.

10. Sakamoto T, Asai Y, Nagao K, Yokota H, Morimura N, Tahara Y, et al. Multicenter non-randomized prospective cohort study of extracorporeal cardiopulmonary resuscitation for out-of hospital cardiac arrest: Study of Advanced Life Support for Ventricular Fibrillation with Extracorporeal Circulation in Japan (SAVE-J). Am Heart Assoc; 2011.

11. Inoue A, Hifumi T, Sakamoto T, Okamoto H, Kunikata J, Yokoi H, et al. Extracorporeal cardiopulmonary resuscitation in adult patients with out-of-hospital cardiac arrest: a retrospective large cohort multicenter study in Japan. Crit Care. 2022;26(1):129.

12. Hifumi T, Inoue A, Takiguchi T, Watanabe K, Ogura T, Okazaki T, et al. Variability of extracorporeal cardiopulmonary resuscitation practice in patients with out-of-hospital cardiac arrest from the emergency department to intensive care unit in Japan. Acute Medicine & Surgery. 2021;8(1):e647.

13. Waljee AK, Mukherjee A, Singal AG, Zhang Y, Warren J, Balis U, et al. Comparison of imputation methods for missing laboratory data in medicine. BMJ Open. 2013;3(8):e002847.

14. Stekhoven DJ, Bühlmann P. MissForest—non-parametric missing value imputation for mixed-type data. Bioinformatics. 2012;28(1):112-8.

15. Moons KG, Altman DG, Reitsma JB, Ioannidis JP, Macaskill P, Steyerberg EW, et al. Transparent Reporting of a multivariable prediction model for Individual Prognosis or Diagnosis (TRIPOD): explanation and elaboration. Ann Intern Med. 2015;162(1):W1-73.

16. Steyerberg EW, Harrell FE, Jr. Prediction models need appropriate internal, internal-external, and external validation. J Clin Epidemiol. 2016;69:245-7.

17. Goto T, Camargo CA, Jr., Faridi MK, Freishtat RJ, Hasegawa K. Machine Learning–Based Prediction of Clinical Outcomes for Children During Emergency Department Triage. JAMA Network Open. 2019;2(1):e186937-e.

18. Parikh RB, Manz C, Chivers C, Regli SH, Braun J, Draugelis ME, et al. Machine Learning Approaches to Predict 6-Month Mortality Among Patients With Cancer. JAMA Network Open. 2019;2(10):e1915997-e.

19. Patel SJ, Chamberlain DB, Chamberlain JM. A Machine Learning Approach to Predicting Need for Hospitalization for Pediatric Asthma Exacerbation at the Time of Emergency Department Triage. Acad Emerg Med. 2018;25(12):1463-70.

20. Levin S, Toerper M, Hamrock E, Hinson JS, Barnes S, Gardner H, et al. Machine-Learning-Based Electronic Triage More Accurately Differentiates Patients With Respect to Clinical Outcomes Compared With the Emergency Severity Index. Ann Emerg Med. 2018;71(5):565-74.e2.

21. Kuhn M, Johnson K, service S. Applied Predictive Modeling. New York, NY: Springer New York : Imprint: Springer; 2013.

22. Package ‘ranger’ [Available from: https://cran.r-project.org/web/packages/ranger/ranger.pdf.

23. Package ‘caret’ [Available from: https://cran.r-project.org/web/packages/caret/caret.pdf.

24. Package ‘xgboost’ [Available from: https://cran.r-project.org/web/packages/xgboost/xgboost.pdf.

25. White AE, Ho AF, Shahidah N, Asyikin N, Liew LX, Pek PP, et al. An essential review of Singapore's response to out-of-hospital cardiac arrests: improvements over a ten-year period. Singapore Med J. 2021;62(8):438-43.
